# Prevalence of noncanonical virulence factors in the adherence and invasion by enterotoxigenic *Escherichia coli* in the small and large intestines

**DOI:** 10.3389/fcimb.2026.1892042

**Published:** 2026-08-24

**Authors:** Karina Espinosa-Mazariego, Sara A. Ochoa, José Arellano-Galindo, Rigoberto Hernández-Castro, Armando Navarro-Ocaña, Sergio Zavala-Vega, Ariadnna Cruz-Córdova, Juan Xicohtencatl-Cortes

**Affiliations:** 1 Laboratorio de Investigación en Bacteriología Intestinal, Unidad de Enfermedades Infecciosas, Hospital Infantil de México Federico Gómez, Mexico City, Mexico; 2 Laboratorio de Virología Clínica y Experimental, Universidad de Investigación en Enfermedades Infecciosas, Hospital Infantil de México Federico Gómez, Mexico City, Mexico; 3 Departamento de Biología Molecular e Histocompatibilidad, Hospital General “Dr. Manuel Gea González”, Mexico City, Mexico; 4 Departamento de Salud Pública, Facultad de Medicina, Universidad Nacional Autónoma de México, Mexico City, Mexico; 5 Laboratorio Clínico y Banco de Sangre, Instituto Nacional de Neurología y Neurocirugía, Mexico City, Mexico; 6 Laboratorio de Investigación en Ciencias Ómicas y Epidemiología Microbiana, Hospital Infantil de México Federico Gómez, Mexico City, Mexico

**Keywords:** adherence, antimicrobial susceptibility, biofilm, enterotoxigenic *Escherichia coli*, invasion, noncanonical virulence factors

## Abstract

**Introduction:**

Enterotoxigenic *Escherichia coli* (ETEC) expresses a diverse set of noncanonical genes that increase its virulence and are involved in mucin degradation, epithelial invasion, and biofilm formation. Collectively, these factors enable effective intestinal colonization, leading to watery diarrhea.

**Aim:**

This study investigated the prevalence of the noncanonical genes *tia*, *tib*A, and *leo*A and their effects on the ability of ETEC to adhere, invade, and form biofilms.

**Materials and methods:**

A total of 244 ETEC strains were analyzed by PCR to confirm the presence of noncanonical virulence genes (*tia*, *tibA*, and *leoA*) and toxins, including heat-labile toxins (LTs) and/or heat-stable toxins (STs). The ability of the strains to adhere to and invade HT-29 colorectal adenocarcinoma cells (large intestine) and HuTu80 duodenal adenocarcinoma cells (small intestine) was evaluated, along with their ability to form biofilms. The susceptibility of these strains to 18 antibiotics was evaluated in accordance with the Clinical & Laboratory Standards Institute (CLSI) 2025 guidelines.

**Results:**

Our data revealed that the *tia*, *tibA* and *leoA* genes were amplified in 11.34% (11/97), 15.46% (15/97), and 7.21% (7/97) of the ETEC strains, respectively. ETEC *tia^+^* strains showed adherence values ranging from 3.6 × 10^5^ to 34.1 × 10^5^ CFUs/mL in HT-29 cells and from 4.1 × 10^5^ to 16.6 × 10^5^ CFUs/mL in HuTu80 cells. ETEC *tib*A*^+^* strains exhibited adherence values ranging from 11.16 × 10^5^ to 65.1 × 10^5^ CFUs/mL in HT-29 cells and from 5.6 × 10^5^ to 27.1 × 10^5^ CFUs/mL in HuTu80 cells. Among the ETEC *tia*^+^ strains, 54.54% (6/11) were invasive, with invasion frequencies ranging 0.17% and 1.01% in HT-29 cells and between 0.20% and 0.404% in HuTu80 cells. Between the strains expressing the *tib*A gene, 47.05% carried both LTs and STs. Among the ETEC *tib*A^+^ strains, 20% were strongly biofilm-producing, 13.33% were moderately biofilm-producing, and 66.66% were weakly biofilm-producing. ETEC *tia^+^/tib*A^+^ strains exhibited high rates of multidrug resistance (MDR).

**Conclusions:**

Our data suggest that noncanonical ETEC strains are highly efficient because their adhesion genes (*tia*, *tib*A, and *leo*A), toxins (*lt* and *st*), and adherence and biofilm-forming capabilities work together to cause infection, even in the absence of some classical virulence factors.

## 1 Introduction

Diarrhea is a leading cause of death worldwide, with more than one million deaths annually (Sang et al., 2025; Kyu et al., 2024). Enterotoxigenic *Escherichia coli* (ETEC) is one of the main bacterial pathogens that causes watery diarrhea in children under five years of age and in elderly individuals (Khalil et al., 2018; Seo et al., 2021). ETEC, the primary etiological agent of traveler diarrhea, also affects military personnel in low- and middle-income countries (Mirhoseini et al., 2018). The World Health Organization (WHO) has indicated that ETEC is responsible for approximately 220 million episodes of diarrhea worldwide, 75 million episodes of diarrhea in children, more than 50,000 deaths of individuals of all ages and 18,700 deaths of children under 5 years of age each year (Khalil et al., 2018; Riaz et al., 2020; World Health Organization, 2021). ETEC is transmitted via the fecal–oral route and is associated with the consumption of contaminated food and water (Gonzales-Siles and Sjöling, 2016). In humans, the dose at which ETEC causes illness ranges from 10^8^ to 10^10^ bacteria (Dubreuil et al., 2016). Furthermore, diarrhea caused by ETEC can vary in severity, from mild and self-limiting to severe with the loss of cholera-like fluid, although it can be fatal in immunocompromised individuals (Morita and Kuroda, 2025). In addition to diarrhea, ETEC infection is often accompanied by vomiting, abdominal pain, headache, and, in some cases, fever (Zhang et al., 2022). Childhood infections caused by this pathogen are particularly concerning because of their associated health complications, including environmental enteropathy, malnutrition, growth retardation, and cognitive impairment, as well as increased susceptibility to irritable bowel syndrome (Troeger et al., 2018; Kuhlmann et al., 2019; World Health Organization, 2021; Zhang et al., 2022).

Genetically, ETEC is a highly heterogeneous pathogen characterized by the presence of plasmids encoding heat-labile toxins (LTs) and/or heat-stable toxins (STs) (Kuhlmann et al., 2019). ETEC has 128 O-antigens and contains 29 colonization factor antigens (CFAs) and coli surface antigenic factors (CSs), both of which are responsible for bacterial colonization of the small intestine in humans and animals (Knutton et al., 1985; Vo et al., 2017; Von Mentzer and Svennerholm, 2023; Morita and Kuroda, 2025). Recent studies have shown that some noncanonical antigens, such as EtpA, EtpB, EatA, Tia, TibA and LeoA, are potentially involved in ETEC pathogenesis (Von Mentzer and Svennerholm, 2023; Vickers et al., 2025). The pathogenesis of ETEC is characterized by its adherence to the small intestine, which is facilitated by various colonization factors, such as fimbriae and *E. coli* surface antigens. Once an ETEC attaches to an enterocyte, it secretes one or both types of LTs and STs. These toxins cause the intestinal epithelium to release fluids, leading to cholera-like diarrhea (Croxen and Finlay, 2009). LTs can be classified into type I or type II, each having distinct genetic, biochemical, and immunological properties (Mirhoseini et al., 2018). STs can be classified into two types: STa and STb (Mirhoseini et al., 2018; Zhang et al., 2022).

Several noncanonical virulence factors play significant roles in the bacterial pathogenicity of ETEC by increasing its virulence. Among these noncanonical virulence factors are autotransporter proteins, which aid in bacterial colonization, survival, and persistence (Henderson and Nataro, 2001; Patel et al., 2004; Vo et al., 2017). ETEC strains are characterized by the presence of one or more of these proteins, including EatA, Tia, and TibA, as well as the two-component secretion systems EtpA and EtpB. EatA is a serine protease encoded on the pCS1 plasmid and belongs to the SPATE family (Patel et al., 2004; Trillo-Muyo et al., 2026). EatA promotes interactions between ETEC and epithelial cells and degrades MUC2, a glycoprotein produced by goblet cells in the small and large intestines, facilitating its entry into epithelial cells and thus promoting infection (Kumar et al., 2014; Kuhlmann et al., 2019; Trillo-Muyo et al., 2026). Other studies have suggested that EatA shares homology with other serine proteases, such as SepA from *Shigella*. Although the function of this protease remains unknown, some studies revealed that this protease accelerated fluid accumulation in an ileal loop model of infection (Kumar et al., 2014).

ETEC strains express the Tia and TibA proteins to adhere to and invade intestinal epithelial cells (Fleckenstein et al., 2010). Tia is a 25 kDa outer membrane protein encoded within a 46 kDa pathogenicity island and interacts specifically with the proteoglycan heparin sulfate. Glycosaminoglycans located on the surface of eukaryotic cells facilitate the initial interaction between the pathogen and host through specific ligand binding (Fleckenstein et al., 2002; Madhavan and Sakellaris, 2015). Tia is homologous to Opa, an outer membrane protein of *Neisseria gonorrhoeae* that mediates cell adhesion and internalization (Fleckenstein et al., 2002), as well as to Hra1 of enteroaggregative *E. coli* (EAEC), which mediates adhesion to and invasion of intestinal cells (Bhargava et al., 2009; Glaubman et al., 2016). TibA is an adhesin with a molecular weight of 104 kDa that is encoded by the *tib* locus. This protein facilitates the colonization of intestinal cells and promotes the formation of bacterial aggregates, serving as a protective mechanism against the host immune system and antimicrobial agents (Vo et al., 2017). TibA shares similarities with the Ag43 protein, which is linked to aggregation, adherence, and intracellular biofilm formation in the bladder epithelium during chronic urinary tract infections (Yao et al., 2014; Vo et al., 2017; Ageorges et al., 2023).

ETEC assembles a two-partner secretion (TPS) system known as EtpABC, which consists of EtpA and EtpB. EtpA is a glycoprotein that plays a crucial role in cell adhesion, whereas EtpB is a pore-forming protein found in the outer membrane (Fleckenstein et al., 2010; Meli et al., 2014*;* Ntui et al., 2023). EtpA has a molecular weight of 170 kDa and interacts with the intestinal epithelium through N-acetylgalactosamine (GalNAc) residues present in mucosal glycoproteins. This interaction promotes adhesion and facilitates the secretion of bacterial toxins (Kuhlmann et al., 2019). Additionally, research has revealed a strong association between EtpA and flagellin (*fli*C), which enhances adhesion to intestinal cells (Fleckenstein et al., 2010; Ntui et al., 2023). EtpB is a 65-kDa outer membrane protein that adopts a β-barrel conformation similar to that of FhaC in *Bordetella pertussis* and acts as an essential component of the TPS, which forms pores to translocate the EtpA adhesin to the cell surface. This process facilitates the intestinal colonization of ETEC (Fleckenstein et al., 2006; Meli et al., 2009). Additionally, ETEC expresses a 100 kDa GTPase called LeoA, which is located on a 46 kb pathogenicity island. This protein is involved in the release of LTs through outer membrane vesicle secretion (Michie et al., 2014). The primary aim of this study was to determine the prevalence of the noncanonical genes *etpA, etpB, eatA, tia, tibA*, and *leoA* in a collection of ETEC strains. Subsequently, specific target lineages (*tia, tibA*, and *leoA*) were selected to correlate the presence of these genes with adherence, invasion and biofilm formation.

## 2 Materials and methods

### 2.1 ETEC bacterial strains

This study involved 244 clinical strains identified as ETEC by serotyping, which were sourced from an *E. coli* culture collection repository at the Facultad de Medicina, Universidad Nacional Autónoma de México, and span the past three decades. All ETEC strains were obtained from stool samples collected from children under five years of age with diarrhea in Mexico (isolated between 1986) and Bangladesh (isolated between 2008 and 2009), as described by Cruz-Córdova et al. (2014) (**Supplementary Table 1**). The ETEC strains utilized in this study were cryopreserved at −70°C immediately following donation. Experimental replicates were prepared to preserve the original isolation passage number and were limited to a maximum of three freeze–thaw cycles during the assays. The strains were initially identified using endpoint polymerase chain reaction (PCR), which amplified genes (*lt, st*, and *lngA*) encoding both LT and ST toxins, as well as the LngA protein associated with the CS21 fimbria (Cruz-Córdova et al., 2014). The ETEC strain H10407 (O78:H11:K80) was used as a positive control to evaluate the presence of noncanonical virulence genes, while the *E. coli* strain DH5α served as a negative control.

### 2.2 Culture media and conditions

The strains were subsequently grown on Luria–Bertani agar (LB, Difco, New Jersey, USA) and incubated at a controlled temperature of 37°C. The bacterial cultures were preserved in 15% glycerol Brain–Heart Infusion broth (BHI, Difco, New Jersey, USA) and stored at -70°C until use. HT-29 colorectal adenocarcinoma cells (ATCC^®^ HTB38™, Manassas, VA, USA) and HuTu80 duodenal adenocarcinoma cells (ATCC^®^ HTB-40™, Manassas, VA, USA) were used for the adherence and invasion assays. HT-29 cells were propagated in Dulbecco’s modified Eagle’s medium (DMEM) (ATCC 30 2002, Manassas, VA, USA), which containing 4.5 g/L glucose supplemented with 10% fetal bovine serum (FBS; ATCC 30-2020; Manassas, VA, USA), and without antibiotics. HuTu80 cells were grown in Eagle’s minimum essential medium (MEM) supplemented with 10% FBS without antibiotics. Both cell lines were detached using a 1X trypsin-Ethylenediaminetetraacetic acid (EDTA) solution (ATCC 302101, Manassas, VA, USA) and incubated at 37°C in an atmosphere containing 5% CO_2_.

### 2.3 Detection of noncanonical virulence genes

The noncanonical virulence genes *etpA*, *etpB*, *eatA*, *leoA*, *tia*, and *tibA* were amplified using PCR under specific conditions, with melting temperatures (TMs) of 49.5°C, 53°C, or 54°C for 25 cycles, as outlined in **Table 1** and detailed in **Supplementary Table 2**. DNA was extracted from the ETEC strains using the Wizard DNA Extraction Kit from Promega (USA). The bacterial strains were cultured overnight in LB broth at 37°C with continuous shaking. PCR assays were conducted using Master Mix PCR from Promega (USA). Following amplification, the DNA products were analyzed by electrophoresis on 1.5% agarose gels (Promega, USA) using 1% TAE (Tris-acetate-EDTA) buffer. The gels were stained with ethidium bromide (5 μg/mL) and visualized using a UV transilluminator (Bio-Imaging Systems, AccesoLab, CDMX, Mexico). ETEC strains positive for *tia*, *tibA*, and/or *leoA* amplification were selected for subsequent phenotypic assays.

**Table 1:** Oligonucleotide sequences, amplicon sizes, and alignment conditions for the PCR detection of noncanonical virulence factors (*etp*A, *etp*B, *eat*A, *tib*A, *tia*, and *leo*A) in ETEC strains.

Gene	Primer	Sequence	Temperature (°C)	No. of Cycles	Size (pb)	Reference
*etp*A	F	GGTTCAGGCAGTATCCAGAC	54	25	999	Del Canto et al., 2011
	R	GGTGTAGCTGTCTGACCACA				Del Canto et al., 2011
*etpB*	F	CTTCGTGCCTGTATCGGTAG	53	25	760	Del Canto et al., 2011
	R	GGTATTTGTCGGTCAGGAAG				Del Canto et al., 2011
*eatA*	F	ATGTGCTTTGGCAGGTTAAT	49.5	25	1943	Del Canto et al., 2011
	R	ATATCCAGTCAGCACCCACT				Del Canto et al., 2011
*tibA*	F	CACGACAAATCAAACGTACC	54	25	655	Del Canto et al., 2011
	R	CTTCCGCCGTAGAGATACAT				Del Canto et al., 2011
*tia*	F	GCTTCAGTTGTGATGCAGAC	54	25	535	Del Canto et al., 2011
	R	CAGCATCCAGATAGCGATAG				Del Canto et al., 2011
*leoA*	F	CGTTTATGGACGATGAGTTG	54	25	574	Del Canto et al., 2011
	R	TCTGCCAGCTCAGTAACAAC				Del Canto et al., 2011

### 2.4 Antimicrobial susceptibility assessment

The antimicrobial susceptibility of ETEC strains carrying the *tia*, *tibA*, and *leoA* genes was evaluated using the Kirby–Bauer diffusion method in accordance with the Clinical and Laboratory Standards Institute (CLSI) guidelines for 2025. A colony was inoculated into Mueller–Hinton (MH) broth (Becton Dickinson, Maryland, USA) from a fresh culture and incubated at 37°C until it reached an OD of 0.5 McFarland. The culture was then spread onto MH agar plates (Becton Dickinson, Maryland, USA), and antibiotic-impregnated discs were placed on the agar. The plates were incubated at 37°C for 18 hours. The *E. coli* strain 25922 (American Type Culture Collection) was used as a control according to the CLSI guidelines. The results were interpreted in accordance with CLSI standards. A total of 18 types of antibiotics were tested: ampicillin (10 μg), amoxicillin/clavulanate (20/10 μg), piperacillin/tazobactam (100/10 μg), ticarcillin/clavulanate (75/10 μg), ceftriaxone (30 μg), ceftazidime (30 μg), cefepime (30 μg), meropenem (10 μg), imipenem (10 μg), aztreonam (30 μg), gentamicin (10 μg), amikacin (10 μg), tetracycline (30 μg), ciprofloxacin (5 μg), nalidixic acid (30 μg), trimethoprim/sulfamethoxazole (1.25/23.75 μg), nitrofurantoin (300 μg), and chloramphenicol (30 μg). All antibiotics were procured from Becton, Dickinson, and Company, Maryland, USA.

### 2.5 HT-29 and HuTu 80 cell adhesion assays

ETEC strains that tested positive for the *tia*, *tibA*, and *leo*A genes were used to assess their ability to colonize intestinal cells through adhesion assays using the HT-29 colorectal adenocarcinoma cell line, which represents the large intestine, and the HuTu80 duodenal adenocarcinoma cell line, which represents the small intestine. For the assays, HT-29 and HuTu80 cells (1 × 10^5^/mL) were seeded into each well of 24-well polystyrene plates (Corning, New York, USA) and incubated overnight at 37°C in a 5% CO_2_ atmosphere. The cells were then infected with overnight bacterial cultures grown in LB broth at a multiplicity of infection (MOI) of 10:1, followed by a 4-hour incubation at 37°C in a 5% CO_2_ atmosphere. After this incubation, nonadherent bacteria were removed by washing the wells three times with 1X PBS. The cell monolayers containing the bacteria were then treated with 0.1% Triton X-100 (Amersham Biosciences, Uppsala, Sweden) for 10 minutes to detach them from the wells. Following several serial dilutions, the samples were plated on LB agar and incubated at 37°C overnight. The bacterial counts were carefully determined as colony-forming units (CFUs) per mL to ensure the accuracy of the results. The adhesion assays were performed in triplicate on three separate days.

### 2.6 Invasion assays using HT-29 and HuTu 80 cells

Monolayers consisting of 80% intestinal cells were infected with ETEC strains and incubated for 4 hours. After the incubation, nonadherent bacteria were removed by washing the cells three times with 1X PBS. Next, DMEM containing 100 µg/ml gentamicin and 100 µg/ml lysozyme was added, and the cells were incubated for an additional 2 hours. Excess antibiotics and lysozyme were then removed by washing the cells with 1X PBS, after which the cells were lysed using 0.1% Triton X-100 (Amersham Biosciences, Uppsala, Sweden) in 1X PBS. Subsequently, 10 µL of the lysates were plated onto LB agar plates and incubated overnight at 37°C. We counted the number of bacteria that grew in the presence of gentamicin and divided it by the total number of CFUs from bacteria that grew without the antibiotic to determine the invasion frequency. Invasion assays were performed in triplicate over three different days.

### 2.7 Biofilm assays using ETEC strains

Biofilm assays were performed using ETEC strains that were confirmed to amplify the *tib*A gene, a specific gene known to mediate biofilm formation, via PCR. The ETEC strains were cultured overnight in Terrific broth (BD DIFCO, New Jersey, USA) with continuous agitation to ensure uniform growth. A suspension was prepared from this bacterial culture and adjusted to an optical density (OD_600 nm_) of 1.0 using Terrific broth. A volume of 200 µL of this suspension was inoculated into each well of a 96-well polystyrene plate, which was subsequently incubated for 48 hours at 37°C to maintain the integrity of the bacterial culture. After the incubation, the medium was carefully removed, and the plate was washed meticulously with 0.85% isotonic saline solution (ISS) before being fixed with 2% formalin. The plates containing the bacterial monolayers were washed three additional times with ISS to ensure the thorough removal of any residual medium. Afterward, they were gently stained with 1% crystal violet. Then, the crystal violet was removed, and the plates were rewashed with ISS. The plates were then allowed to dry, and the crystal violet was solubilized using 70% methanol. Biofilm formation was quantified by measuring the OD at 600 nm, which is directly correlated with the amount of eluted crystal violet. Importantly, these biofilm assays were conducted in triplicate across three independent experiments to ensure the reproducibility of the results. The absorbance of Terrific broth was used to classify biofilm formation into four categories—nonbiofilm formers and weak, moderate, or strong biofilm formers—based on the OD_600nm_ of bacterial films (Stepanovic et al., 2000). The ETEC 114191 strain was used as a negative control and has previously been described as a non-biofilm-forming strain by Cruz-Córdova et al. (2014). The cutoff OD_600_ value for a 96-well microplate was defined as two standard deviations above the mean OD_600nm_ value of the negative control.

### 2.8 Statistical analysis

The Mann–Whitney U test was used to compare bacterial adherence and invasion between HT-29 and HuTu 80 cells. For each cell line, bacterial adherence and invasion were evaluated using the Kruskal–Wallis (H) test, followed by Dunn’s *post hoc* multiple comparisons test. Statistical significance was defined as a p value less than 0.05. Analyses were performed using GraphPad Prism, version 8.0.0 for Windows (GraphPad Software, San Diego, California, USA).

## 3 Results

### 3.1 Prevalence and cooccurrence of noncanonical virulence genes in ETEC strains

Our study revealed that at least one noncanonical virulence gene was amplified in 39.75% (97/244) of the previously characterized ETEC strains. Among these ETEC strains, the *etpA* and *etpB* genes were amplified in 75.25% (73/97), *eatA* was amplified in 56.7% (55/97), *tibA* was amplified in 15.46% (15/97), *tia* was amplified in 11.34% (11/97), and *leoA* was amplified in 7.21% (7/97).

The ETEC *tia*^+^ strains presented the following frequencies with other noncanonical genes: 72.72% (8/11) harbored the *etp*A gene, 72.72% (8/11) harbored the *etp*B gene, 27.27% (3/11) harbored the *eat*A gene, 9.09% (1/11) harbored the *tib*A gene, and 63.63 (7/11) harbored the *leo*A gene; however, 27.27% (3/11) of the samples did not show any combination with these genes. Among the ETEC strains in which the expression of the *tib*A gene and noncanonical genes was amplified, 73.33% (11/15) expressed the *eat*A, *etp*A, and *etp*B genes. Additionally, the *tia* gene was detected in 6.66% (1/15) of the strains, while 26.66% (4/15) did not show any combination with other noncanonical genes (**Supplementary Figure 1**).

### 3.2 ETEC strains *tia^+^*, *tib*A^+^, and *leoA*^+^ showed resistance to several antibiotic types

**Figure 1:**
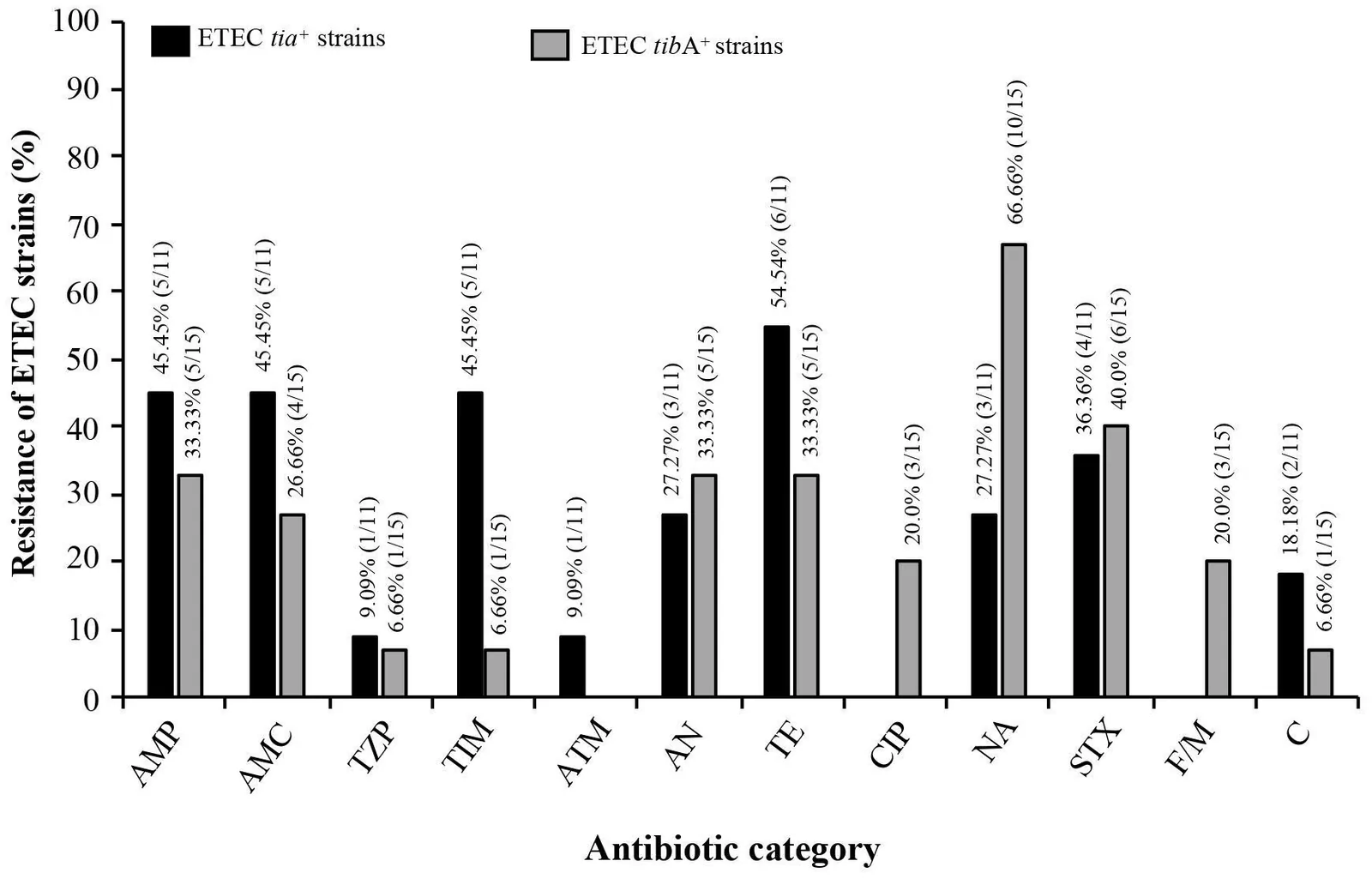
Resistance profiles of noncanonical ETEC strains carrying the *tia* and *leo*A genes. Percentages of strains resistant to the following antibiotics: ampicillin (AMP), amoxicillin/clavulanate (AMC), piperacillin/tazobactam (TZP), ticarcillin/clavulanate (TIM), aztreonam (ATM), amikacin (AN), tetracycline (TE), ciprofloxacin (CIP), nalidixic acid (NA), trimethoprim/sulfamethoxazole (STX), nitrofurantoin (F/M), and chloramphenicol (C).

In this study, 11 ETEC *tia^+^* strains, 15 ETEC *tib*A^+^ strains, and seven ETEC *leo*A^+^ strains were tested for resistance to different antibiotic categories (**Tables 2**, **3**). Interestingly, 63.63% (7/11) of the ETEC *tia*^+^ strains exhibited multidrug-resistance (MDR) profiles (**Table 2**). In addition, 54.54% (6/11) of the ETEC *tia*^+^ strains were resistant to tetracycline, whereas 45.45% (5/11) demonstrated consistent resistance to ampicillin, amoxicillin/clavulanic acid, and ticarcillin/clavulanic acid (**Figure 1**). In contrast, 36.36% (4/11) were resistant to trimethoprim/sulfamethoxazole, and 9.09% (1/11) were resistant to aztreonam. In contrast, 100% of ETEC *tia*^+^ strains were susceptible to ceftriaxone, ceftazidime, cefepime, meropenem, imipenem, gentamicin, and nitrofurantoin (**Table 2**).

**Table 2:** Analysis and genetic correlation of ETEC strains harboring the *tia* gene, along with the cooccurrence of other noncanonical virulence genes and toxins.

		Genes				Category of antibiotics												
Strains	*tia*	*lt*	*st*	*tibA*	*leoA*	Penicillin	β- lactams	Cephalosporins III	Cephalosporins IV	Carbapenems	Monobactam	Lipopeptides	Tetracyclines	Fluoroquinolones	Quinolones	Folates	Nitrofurans	Phenicol
114120	+	–	+	–	+	R	R	S	S	S	S	S	R	S	R	S	S	S
114126	+	–	+	–	+	R	R	R	S	S	R	S	R	S	R	R	S	S
114150	+	+	+	–	–	S	S	S	S	S	S	S	R	S	R	R	S	S
114188	+	–	+	–	+	S	S	S	S	S	S	R	S	S	R	S	S	S
114202	+	+	+	–	–	R	R	S	S	S	S	S	R	S	R	R	S	R
114208	+	+	–	–	+	S	S	S	S	S	S	S	S	S	R	S	S	S
114216	+	+	–	–	+	R	S	S	S	S	S	S	R	S	R	S	S	S
114218	+	+	–	–	+	S	S	S	S	S	S	S	S	S	R	S	S	S
114219	+	+	+	–	–	S	R	S	S	S	S	R	S	S	R	R	S	R
114225	+	+	–	–	+	S	S	S	S	S	S	S	R	S	R	S	S	S
115311	+	–	+	+	–	R	R	S	S	S	S	R	S	S	R	R	S	S

**Table 3:** Analysis and genetic correlation of ETEC strains harboring the *tib*A gene, along with the cooccurrence of other noncanonical virulence genes and toxins.

		Genes				Category of antibiotics												
Strains	*tibA*	*lt*	*st*	*tia*	*leoA*	Penicillins	β- lactams	Cephalosporins lll	Cephalosporins lV	Carbapenems	Monobactam	Lipopeptides	Tetracyclines	Fluoroquinolones	Quinolones	Folates	Nitrofurans	Phenicols
44166	+	–	+	–	–	S	S	S	S	S	S	S	S	S	S	S	S	R
45162	+	+	+	–	–	R	S	S	S	S	S	R	S	S	S	S	S	S
61162	+	+	–	–	–	S	S	S	S	S	S	S	S	S	S	S	S	S
63280	+	–	+	–	–	S	S	S	S	S	S	S	S	S	S	S	R	S
63284	+	–	+	–	–	R	S	S	S	S	S	S	S	S	R	S	S	S
115311	+	–	+	+	–	R	R	S	S	S	S	R	S	S	R	R	S	S
115340	+	+	+	–	–	S	R	S	S	S	S	R	R	S	R	R	R	S
115341	+	+	+	–	–	S	S	S	S	S	S	R	R	S	R	R	S	S
115342	+	+	+	–	–	S	R	S	S	S	S	R	R	S	R	R	R	S
115343	+	+	–	–	–	S	S	S	S	S	S	S	S	S	R	R	S	S
115345	+	+	+	–	–	R	S	S	S	S	S	S	S	R	R	R	S	S
115346	+	+	+	–	–	S	S	S	S	S	S	S	R	R	R	S	S	S
115363	+	+	–	–	–	R	S	S	S	S	S	S	S	R	R	S	S	S
115364	+	–	+	–	–	S	R	S	S	S	S	S	S	S	R	S	S	S
115368	+	+	+	–	–	S	S	S	S	S	S	S	S	S	S	S	S	R

Penicillins (ampicillin, AMP), β-lactams (amoxicillin/clavulanate, AMC; piperacillin/tazobactam, TZP; ticarcillin/clavulanate, TIM), third-generation cephalosporins (ceftriaxone, CRO; ceftazidime, CAZ), fourth-generation cephalosporins (cefepime, FEP), carbapenems (meropenem, MEM; imipenem, IMP), monobactams (aztreonam, ATM), lipopeptides (gentamicin, GM; amikacin, AN), tetracyclines (tetracycline, TE), fluoroquinolones (ciprofloxacin, CIP), quinolones (nalidixic acid, NA), folate pathway inhibitors (trimethoprim/sulfamethoxazole, STX), nitrofurans (nitrofurantoin, F/M), and phenicols (Chloramphenicol, C).

Additionally, three ETEC strains positive for both the *leoA* and *tia* genes were categorized as MDR, accounting for 42.85% (3/7) (**Table 2**). Among the ETEC *tib*A^+^ strains, 46.66% (7/15) were classified as MDR. Within this group, resistance to nalidixic acid was most prevalent and was observed in 66.66% (10/15) of the strains, whereas resistance to trimethoprim/sulfamethoxazole was observed in 40% (6/15) of the strains (**Table 3**). Furthermore, 33% (5/15) exhibited simultaneous resistance to ampicillin, amikacin, and tetracycline (**Table 3**). Interestingly, resistance to ciprofloxacin and nitrofurantoin was detected in 20% (3/15) of the ETEC *tib*A^+^ strains, whereas none of the ETEC *tia^+^* strains exhibited resistance (**Figure 1**). Notably, all the ETEC *tib*A^+^ strains were 100% susceptible to ceftriaxone, ceftazidime, cefepime, meropenem, imipenem, aztreonam, and gentamicin (**Table 3**).

### 3.3 ETEC strains with *tia*, *tib*A, and *leoA* genes were capable of adhering to HuTu80 and HT-29 cells

**Figure 2:**
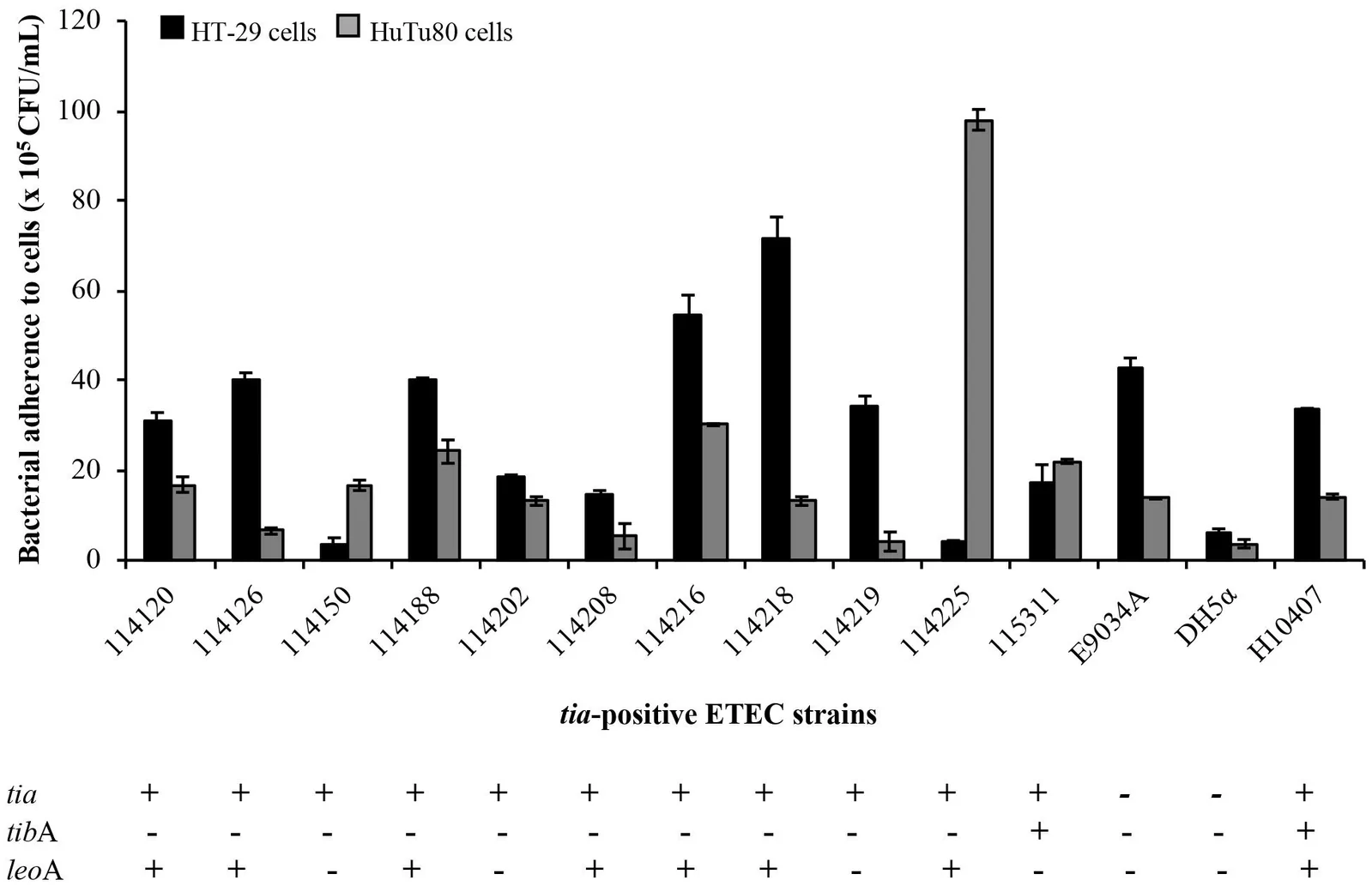
Adherence profile of ETEC *tia*^+^ strains to HT-29 and HuTu80 cells. ETEC *tia*^+^ strains exhibit considerable variability in their ability to adhere to HT-29 and HuTu80 cells. These findings confirm that this outer membrane protein functions as an effective adhesin in both the colonic and duodenal epithelia.

The tested ETEC strains included those with amplification of the *tia* gene, either alone or in combination with *tib*A and *leo*A. ETEC strain 114218 exhibited the greatest adherence to HT-29 cells (71.55 x 10^5^ UFCs/mL), whereas ETEC 114225 displayed the greatest adherence to HuTu80 cells (98.0 x 10^5^ UFCs/mL). Additionally, three ETEC (114150, 114202, and 114219) strains with the *tia^+^/tib*A^-^/*leo*A^-^ genotype presented adherence values ranging from 3.66 × 10^5^ CFUs/mL to 34.11 × 10^5^ CFUs/mL in HT-29 cells and from 4.15 × 10^5^ CFUs/mL to 16.66 × 10^5^ CFUs/mL in HuTu80 cells (**Figure 2**). The ETEC 115311 *tia^+^/tib*A^+^/*leo*A^-^ strain exhibited adherence levels of 15.8 × 10^5^ CFUs/mL to HT-29 cells and 17.5 × 10^5^ CFUs/mL to HuTu80 cells (**Figure 2**). ETEC (114120, 114126, 114188, 114208, 114216, 114218, and 114225) *tia^+^/tib*A^-^/*leo*A^+^ strains showed varying levels of adherence to HT-29 and HuTu80 cells. The adherence to the HT-29 cells ranged from 3.66 × 10^5^ CFUs/mL to 71.55 × 10^5^ CFUs/mL, whereas the adherence to the HuTu80 cells ranged from 4.22 × 10^5^ CFUs/mL to 98.0 × 10^5^ CFUs/mL (**Figure 2**).

ETEC *tia*^+^ strains exhibited significantly greater adherence to HT-29 cells than to HuTu80 cells (U = 0, *P* < 0.0001), except for strains 114150 and 114225 (U = 0, *P* < 0.0001) and strain 115311 (U = 14.5, P = 0.0144), which showed greater adherence to HuTu80 cells. Significant differences in the percentages of adherent ETEC strains were observed in HT-29 cells (H = 122.5, *P* < 0.0001, n = 126). Dunn’s *post hoc* multiple comparison test revealed that strains 114126, 114188, 114216, 114218, and E9034A exhibited high adherence to HT-29 cells, whereas strains 114150, 114202, 114208, 114225, and DH5α displayed low adherence. The results of the Kruskal–Wallis test also revealed significant differences in adherence among the ETEC strains in HuTu80 cells (H = 117.4, *P* < 0.0001, and n = 126). ETEC strains 114225, 114216, and 114188 exhibited the highest adherence, whereas strains 114126, 114208, and 114219 showed the lowest adherence. Notably, the adherence of the ETEC strains 114216 and 114188 to both cell lines was significantly high.

**Figure 3:**
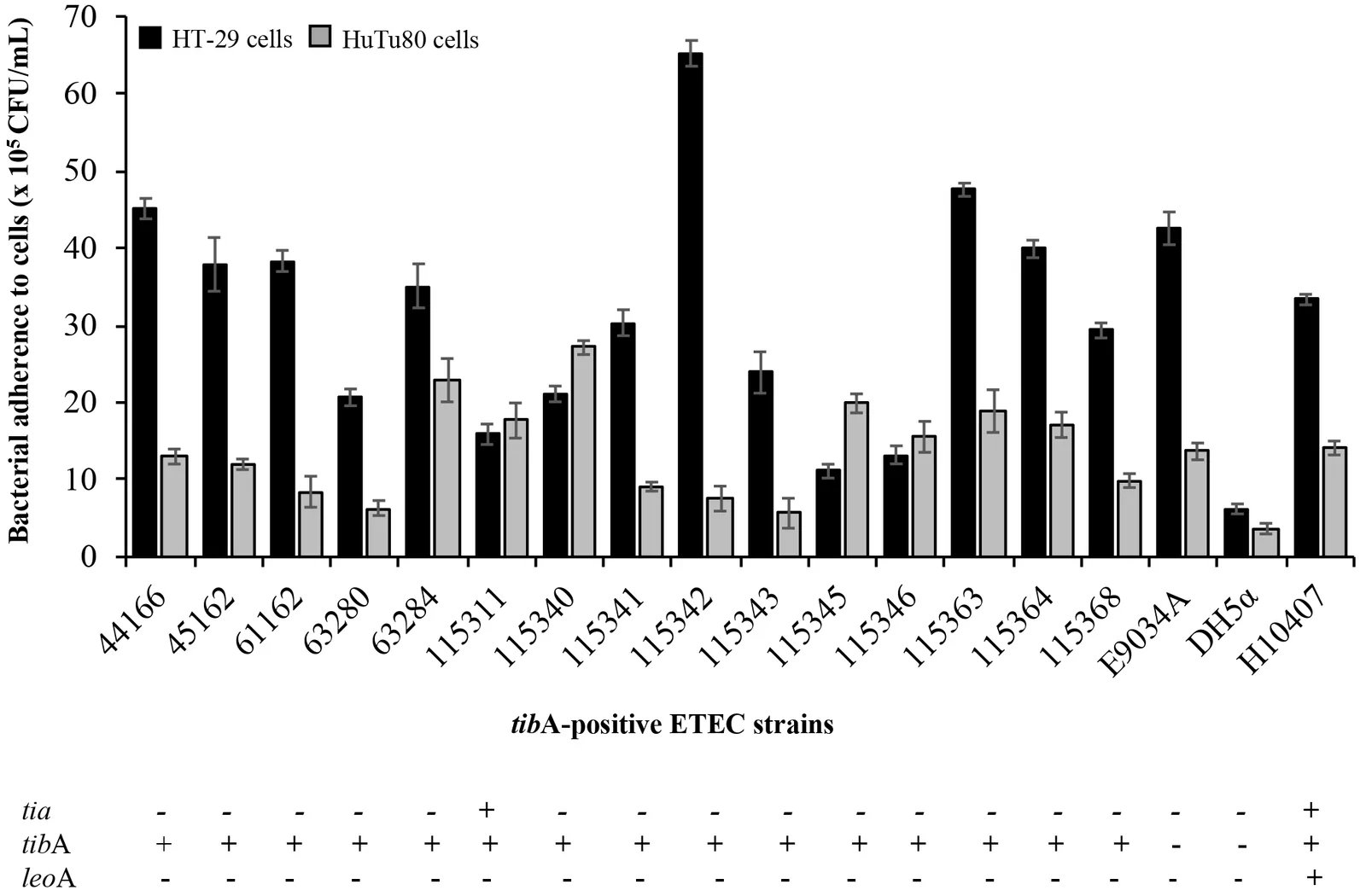
Adherence profile of the ETEC *tib*A^+^ strains to the HT-29 and HuTu80 cells. Phenotypic variability in bacterial adherence to the epithelial cells of the colon and duodenum is mediated by the autotransporter glycoprotein *tib*A.

Moreover, 14 ETEC strains containing the *tib*A gene but lacking a relationship with *tia* and *leo*A exhibited varying levels of adherence to the cell lines HT-29 and HuTu80. The adherence values of these strains to HT-29 cells ranged from 11.16 × 10^5^ CFUs/mL to 65.16 × 10^5^ CFUs/mL, whereas the adherence to HuTu80 cells ranged from 5.66 × 10^5^ CFUs/mL to 27.16 × 10^5^ CFUs/mL (**Figure 3**). Among these strains, strain 115340 exhibited the highest adherence to HuTu80 cells, whereas strain 115342 had the highest adherence to HT-29 cells (**Figure 3**). In contrast, the ETEC strain 115345 exhibited minimal adherence to HT-29 cells and 115343 to HuTu80 cells (**Figure 3**).

The adherence of ETEC *tibA*^+^ strains to HT-29 cells was significantly higher than that to HuTu80 cells (U = 0, *P* < 0.0001). However, ETEC strains 115340 and 115345 (U = 0, *P* < 0.0001) and 115346 (U = 14, *P* = 0.0144) exhibited greater adherence to HuTu80 cells. ETEC strain 115311 (U = 28.5, *P* = 0.3026) showed comparable adherence to both cell lines. In HT-29 cells, significant differences in the adherence capacity were observed among these ETEC strains (H = 166.9, *P* < 0.0001, n = 171). Dunn’s *post hoc* multiple comparison test indicated that the adherence of the ETEC strains 44166, 115342, 115363, 115364, and E9034A to HT-29 cells was significantly greater, whereas the adherence of the ETEC strains 115345, 115346, 115311, 115322, and DH5α to these cells was low. In HuTu80 cells, significant differences in adherence were also observed among these ETEC strains (H = 152.5, *P* < 0.0001, and n = 162). *Post hoc* tests revealed high adherence of the ETEC strains 115340, 63284, 115345, 115311, 115363, and 115364. ETEC strains 61162, 63280, 115342, 115343, 115341, and DH5α exhibit low adherence.

### 3.4 ETEC strains harboring *tia^+^*, *tib*A^+^, and *leoA*^+^ genes were able to invade HT-29 and HuTu80 cells

**Figure 4:**
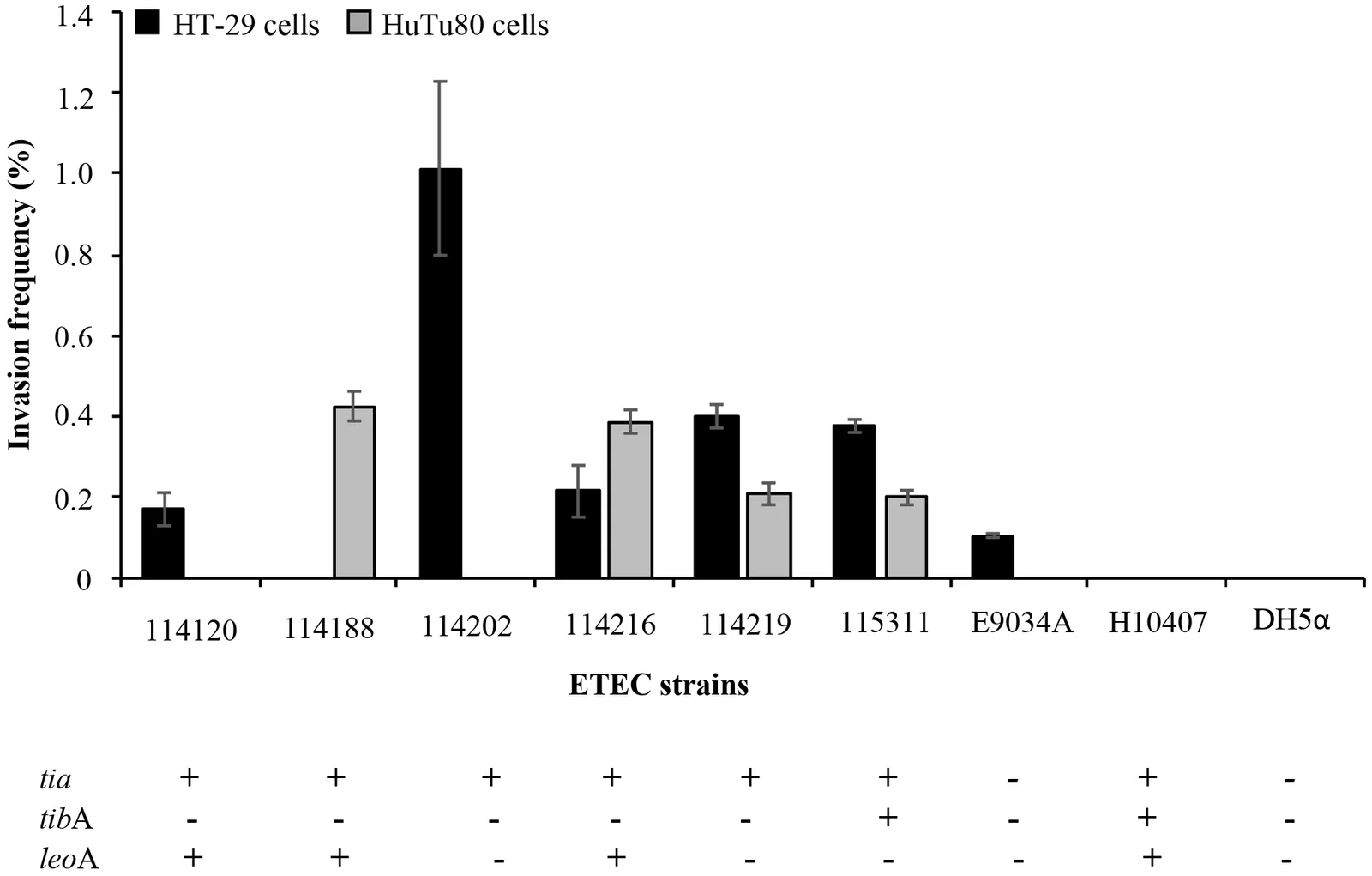
Frequency of invasion of ETEC *tia*^+^ strains into HT-29 and HuTu80 cells. ETEC strains carrying the *tia* and *leo*A genes exhibit variable internalization rates in both HT-29 and HuTu80 cells, confirming that this protein functions in the invasion of both colonic and duodenal cells.

In this study, we assessed the invasion frequency of ETEC *tia*^+^ strains harboring one, both, or neither the *tib*A nor the *leo*A gene. In addition, 54.54% (6/11) of the ETEC strains expressing the *tia* gene exhibited varying invasion rates in HT-29 and/or HuTu80 cells (**Figure 4**). Specifically, the invasion frequencies of the ETEC (114120 and 114216) *tia*^+^/*leo*A^+^ strains were 0.17% and 0.21%, respectively, in the HT-29 cells (**Figure 4**). In addition, the invasion frequencies of the ETEC (114188 and 114216) *tia*^+^/*leo*A^+^ strains were 0.404% and 0.37%, respectively, in HuTu80 cells (**Figure 4**). On the other hand, the invasion frequencies of the ETEC (114202 and 114219) *tia*^+^/*tib*A^-^/*leo*A^-^ strains were 1.01% and 0.401%, respectively, in the HT-29 cells (**Figure 4**). The invasion frequency of the ETEC 114219 *tia*^+^/*tib*A^-^/*leo*A^-^ strain was 0.201% in HuTu80 cells (**Figure 4**). The invasion frequency of the ETEC 115311 *tia*^+^/*tib*A^+^/*leo*A^-^ strain was 0.37% in the HT-29 cells and 0.20% in the HuTu80 cells (**Figure 4**). Conversely, the ETEC 114126 *tia*^+^/*tib*A^-^/*leo*A^+^ strain, ETEC 114150 *tia*^+^/*tib*A^-^/*leo*A^-^ strain, ETEC 114208 *tia*^+^/*tib*A^-^/*leo*A^+^ strain, ETEC 114218 *tia*^+^/*tib*A^-^/*leo*A^+^ strain, and ETEC 114225 *tia*^+^/*tib*A^-^/*leo*A^+^ strain did not invade HT-29 or HuTu80 cells.

A comparative analysis employing the Mann–Whitney U test revealed three strains that exhibited an invasive phenotype in both cell lines (U = 0, *P* = 0.0022). Quantification of the degree of invasiveness of the ETEC strains in the HT-29 cells using the Kruskal–Wallis rank-sum test revealed highly significant heterogeneity (H = 32.57, *P* < 0.0001, and n = 36). The *post hoc* analysis indicated that compared with the other ETEC strains, strain 114202 exhibited the highest invasion index, with statistically significant differences. In contrast, the ETEC strains 114120, 114216, and E90934A did not differ significantly from each other, similar to the behavior observed for strains 114219 and 115311. Finally, compared with strains 114219 and 115311, strain 114188 had a significantly greater invasion capacity in HuTu 80 cells (*P* < 0.001). Statistical analysis using the Mann–Whitney U test revealed a lower invasion frequency by ETEC strains in HuTu 80 cells.

### 3.5 Relationships between toxin-encoding genes and noncanonical virulence genes in ETEC strains

The genotypes of the 11 ETEC *tia*^+^ strains were categorized as follows: 63.63% (7/11) of the strains carried *tia*^+^/*leo*A^+^, 36.36% (4/11) of the strains carried *tia*^+^/*leo*A^-^, and 9.0% (1/11) of the strains carried *tia*^+^/*tib*A^+^/*leo*A^-^ (**Table 2**). The alleles *lt* and/or *st* were distributed heterogeneously among ETEC *tia*^+^ strains either alone or in combination with the *tib*A and *leo*A genes. Specifically, 63.63% (7/11) of these ETEC strains harbored the *lt* gene, whereas the remaining ETEC strains harbored the *st* gene (**Table 2**). The *lt* gene was present in 36.36% (4/11) of the ETEC *tia*^+^/*tib*A^-^/*leo*A^+^ strains, whereas the *st* gene was detected in 27.27% (3/11) of the ETEC *tia*^+^ *tib*A^-^//*leo*A^+^ strains. Intriguingly, 27.27% (3/11) of the ETEC *tia*^+^/*tibA*^-^/*leo*A^-^ strains possessed both the *lt* and *st* alleles. Conversely, the *tia*^+^/*tib*A^+^/*leo*A^-^ strain expressed only the *st* gene (**Table 2**). Additionally, among the 15 ETEC *tib*A*^+^* strains with the *tia*^-^/*leo*A^-^ genotype, 20% (3/15) amplified the *lt* gene alone, 33.33% (5/15) amplified the *st* gene alone, and 46.66% (7/15) amplified both the *lt* and *st* genes (**Table 3**). Cooccurrence was observed with *tib*A*^+^* and *tia*^+^ in one ETEC strain.

### 3.6 ETEC strains carrying the *tibA* gene showed the ability to form biofilms

Several studies have shown that ETEC strains carrying the *tib*A gene are primarily involved in a high capacity for biofilm formation. A total of 15 *tib*A-positive ETEC strains were included in the biofilm assays. Among these strains, 20% (3/15) were strong biofilm formers, with OD_600nm_ values ranging from 0.231 to 0.242 (**Table 4**). Additionally, 13.33% (2/15) were classified as moderate biofilm formers, with OD_600nm_ values ranging from 0.132 to 0.153, and 66.66% (10/15) were classified as weak-biofilm-producing strains, with OD_600nm_ values ranging from 0.061 to 0.109 (**Table 4**). ETEC H10407 was used as a positive control and was classified as a moderate biofilm former. Terrific broth was used as a negative control. The classifications were based on the criteria established by Stepanovic et al., 2000: strains lacking the ability to produce biofilms (nonadherent) have an OD_600nm_ of 0 or less (OD ≤ ODc), those with a weak biofilm-forming ability have an OD_600nm_ greater than 0 but less than or equal to 2 (ODc < OD ≤ 2 ODc), those with a moderate ability to produce biofilms have an OD_600nm_ greater than 2 but less than or equal to 4 (2 X ODc < OD ≤ 4 ODc), and those with a strong ability to produce biofilms have an OD_600nm_ greater than 4 (4 X ODc < OD) (**Table 4**).

**Table 4:** Biofilms of ETEC strains in which the *tib*A gene was amplified. In accordance with Srdjan Stepanovic’s criteria, the biofilm formation of ETEC strains in which the *tibA* gene was amplified was classified as weakly biofilm-forming for those with OD values between 0.057 and 0.114, moderately biofilm-forming for those with OD values from 0.114 to 0.228, and strongly biofilm-forming for those with OD values equal to or greater than 0.228.

Strains	Biofilm values OD_600nm_	Standard deviation (SD)	Biofilms (forming)
44166	0.242	0.023	Strong
45162	0.132	0.019	Moderate
61162	0.153	0.005	Moderate
63280	0.090	0.001	Weak
63284	0.065	0.005	Weak
115311	0.082	0.019	Weak
115340	0.063	0.001	Weak
115341	0.104	0.001	Weak
115342	0.234	0.027	Strong
115343	0.109	0.005	Weak
115345	0.072	0.007	Weak
115346	0.082	0.005	Weak
115363	0.080	0.022	Weak
115364	0.061	0.001	Weak
115368	0.231	0.035	Strong
H10407	0.226	0.006	Moderate
114191	0.056	0.002	No
Terrific Broth	0.057	0.001	No

## 4 Discussion

Adherence to intestinal cells is a crucial initial step in infection by ETEC strains. This pathogen produces several CFAs, including CS21 and CS3, as well as other proteins known as noncanonical factors, such as *etp*A, *etp*B, *eat*A, *tia*, *tib*A, and *leo*A, that have been understudied (Del Canto et al., 2011). Among the 244 ETEC strains examined in our study, 56.7% amplified the *eat*A gene. A study involving ETEC strains associated with traveler diarrhea among Spanish travelers reported that 48% of these strains tested positive for the *eatA* gene (Rivera et al., 2012).

Our data revealed a low prevalence of the genes *tia* (11.34%) and *leo*A (7.21%). These findings are consistent with those of previous studies, which reported frequencies of 8.7% for *tia* and 5.8% for *leo*A (Del Canto et al., 2011). The strong presence between *tia* and *leo*A (63.63%) is biologically relevant, as both genes are located in the same genomic region on the Tia-PAI pathogenicity island (Del Canto et al., 2011). This genomic cooccurrence in specific ETEC clonal lineages results from the structural conservation of the *tia* locus adjacent to the intrachromosomal *leo*BC operon (Michie et al., 2014). Comparative genomic studies have revealed that the “split” organization of *leo*BC is present in various bacteria and that its proximity to *tia* is a distinctive characteristic of ETEC.

This study revealed that the *tib*A gene was present in 15.46% of the positive ETEC strains. These findings contrast with those of prior epidemiological studies reporting a lower prevalence of 10.7% (Del Canto et al., 2011). This significant difference suggests that these ETEC strains may exhibit greater adhesin diversity or originate from geographic areas where this virulence factor confers a distinct fitness advantage under localized selective pressure. Similarly, TibA and other autotransporters, such as *sat*, are associated with prolonged diarrhea or persistent infections because of their ability to evade the immune system and resist mechanical elimination in the digestive tract (Zude et al., 2013).

Our data indicate that strains harboring *tia* (63.63%) genes exhibit high rates of MDR, particularly to tetracycline (54.54%), ampicillin (45.45%), amoxicillin–acid clavulanate (45.45%), ticarcillin/clavulanate (45.455), and trimethoprim/sulfamethoxazole (36.36%). In addition, high rates of MDR were detected among the *tib*A-positive ETEC strains, particularly to nalidixic acid (66.66%), trimethoprim/sulfamethoxazole (40.0%), amikacin (33.33%), and tetracycline (33.33%). These findings are consistent with previously reported data (Fatema-Tuz et al., 2024; Begum et al., 2026). Notably, 66.66% of the *tib*A-positive strains exhibit resistance to nalidixic acid, likely because of mutations in the *gyr*A gene (Yang et al., 2021). Fortunately, unlike ESBL-producing ETEC strains, all ETEC strains show 100% susceptibility to carbapenems and cephalosporins (Joffré et al., 2025). In our studies, a differential tropism to both cell lines (HT-29 cells and HuTu80 cells) was observed among the 14 strains that exclusively harbor the *tib*A^+^ gene, with a preferential affinity for HT-29 cells (up to 65.16 × 10^5^ CFUs/mL) and a lower affinity for HuTu80 cells (up to 27.16 × 10^5^ CFUs/mL). Interestingly, the adherence of the ETEC *tia*+ strains was high for both the HT-29 cells (up to 71.55 × 10^5^ CFUs/mL) and the HuTu80 cells (up to 98 × 10^5^ CFUs/mL). These findings suggest that Tia functions independently as an afimbrial adhesin and is an important factor for the colonization of ETEC strains (Mammarappallil and Elsinghorst, 2000). Consistent with these observations, previous studies have shown the *tia* gene alone is sufficient to confer an adherence phenotype in laboratory strains, even in the absence of other factors (Elsinghorst and Weitz, 1994). The *tib*A gene typically serves as the primary effector when both the *tia* and *tibA* genes are present (Elsinghorst and Weitz, 1994). Previous studies have shown that compared with the wild-type strain, the deletion of the *tib* locus leads to an approximately 75% decrease in adherence (Lindenthal and Elsinghorst, 2001). Even if a strain lacks *tib*A, the *tia* gene alone provides most of the binding energy required for intimate contact (Fleckenstein et al., 1996). Unlike fimbriae (CFA/I), which mediate weak and reversible initial attachment, electron microscopy studies have shown that Tia induces a “zipper” interaction (Tollis et al., 2010). This interaction is characteristic of ETEC strains that utilize noncanonical adhesins or CFAs (Del Canto et al., 2012; Rodríguez-Martínez et al., 2025).

Our data revealed that the interaction rates of the ETEC strains with HuTu80 cells were as high as 98 × 10^5^ CFUs/mL, consistently surpassing the adherence rates observed for the HT-29 cells, which reached 71.55 × 10^5^ CFUs/mL. These findings suggest that compared with HT-29 cells, ETEC has a greater ability to colonize the small intestine and bind more strongly to HuTu80 cells. This pattern of adherence is consistent with the findings from other studies that indicate that the coordinated expression of these genes enhances bacterial interactions with cells in the upper intestinal epithelium, particularly in strains that possess both genes. The *tia* locus often includes the *leo*A gene, which encodes a protein that directly interacts with enterocytes. The *leo*A gene facilitates the efficient release of LTs via extracellular vesicles, leading to increased cell damage and the formation of a feedback loop that stabilizes bacterial adherence (Mammarappallil and Elsinghorst, 2000; Kesty et al., 2004; Michie et al., 2014; Rodríguez-Martínez et al., 2025).

The combined presence of the *tia* and *leo*A genes appeared to significantly increase the colonization capacity of the ETEC114218 strain on HT-29 cells, with adherence reaching 71.55 × 10^5^ CFUs/mL. However, ETEC strain 11425 which also possesses the *tia* and *leo*A genes, displayed the lowest adhesion to HT-29 cells (3.88 × 10^5^ CFUs/mL). These findings align with previous studies indicating considerable variability in adhesion capabilities among clinical *E. coli* isolates *in vitro* (Mammarappallil and Elsinghorst, 2000; Sharma and Kanwar, 2017). Furthermore, prior studies have shown that the *tia* locus not only acts as an adhesin but also contributes to an invasive phenotype in cell lines such as HCT-8 and HT-29 (Mammarappallil and Elsinghorst, 2000; Brown and Hardwidge, 2007; Mudrak and Kuehn, 2010; Michie et al., 2014). These data are consistent with those of previous studies indicating that various clinical *E. coli* isolates can exhibit a wide range of adhesion capabilities *in vitro* (Mammarappallil and Elsinghorst, 2000; Sharma and Kanwar, 2017). Our data revealed that the ETEC 114225 strain had the greatest adhesion to HuTu-80 cells (98 × 10^5^ CFUs/mL), whereas strain 114208 exhibited the lowest adhesion (5.3 × 10^5^ CFUs/mL). Our data revealed that ETEC strains with the *tibA* gene (but lacking *tia* and *leoA*) exhibit a significant and variable adherence capacity. Furthermore, ETEC 115340 showed greater adherence to HuTu80 cells, whereas ETEC 115342 showed greater adherence to HT-29 cells. In contrast, the adherence of the ETEC strain 115345 to HT-29 and HuTu80 cells was low. The variability in adherence observed between HuTu80 and HT-29 cells suggests that adhesins such as TibA can interact with different surface receptors depending on the cell type or expression of other fimbria, such as those of the CS21 pilus, which explains why the same strain does not perform equally well in both cell lines (Darfeuille-Michaud et al., 1990; Kerneis et al., 1994; Saldaña-Ahuactzi et al., 2016; Rodríguez-Martínez et al., 2025).

Our study revealed that 36.36% of the ETEC *tia^+^*/*leo*A^-^ strains demonstrated varying but detectable invasion rates in HT-29 and/or HuTu80 cells. Based on the adherence properties of ETEC, the genes *tib*A and *leo*A play significant roles in mediating interactions with host cells (Elsinghorst and Weitz, 1994; Del Canto et al., 2011; Michie et al., 2014). Studies involving strain H10407 have shown that the *tia* gene alone can promote invasion in cell lines such as HCT-8 (Mammarappallil and Elsinghorst, 2000). The presence of the *leo*A gene, which is often located within the *tia* locus in pathogenicity islands, helps explain why our ETEC strains carry these genes simultaneously and exhibit invasive characteristics (Del Canto et al., 2011; Gonzales et al., 2013; Michie et al., 2014). The ETEC strains exhibited significantly high invasion rates in HT-29 cells; specifically strains 114202 and 114120 exhibited invasion rates of 1.01% and 0.17%, respectively. In contrast, other studies using strain H10407 in intestinal cells typically report invasion levels ranging from 0.1% to 0.5%. These findings identify 114202 as an ETEC strain demonstrating exceptional efficiency in cellular invasion (Elsinghorst and Kopecko, 1992; Fleckenstein et al., 1996; Mammarappallil and Elsinghorst, 2000; Fleckenstein et al., 2014).

According to our data, the 0.42% invasion frequency observed for strain 114188 in HuTu80 cells aligns with previous studies reporting similar invasion frequencies for strain H10407 and other ETEC strains that are positive for the *tia* locus across other cell lines (Fleckenstein et al., 1996; Mammarappallil and Elsinghorst, 2000). The variation in invasion frequency could be due to differences in the expression or glycosylation levels of the TibA protein (Elsinghorst and Weitz, 1994; Sherlock et al., 2005). The increased invasion observed in HT-29 cells (up to 1.01%) compared with HuTu80 cells (0.42%) suggests that the receptors for Tia and TibA may be more accessible or more abundant in colonic cells (HT-29 cells) than in duodenal cells (HuTu80 cells). However, gene expression assays are needed to confirm this hypothesis. The *tia* gene is homologous to the *Yersinia ail* locus, reinforcing its classification as a highly specialized determinant of cell invasion (Fleckenstein et al., 1996).

Molecular characterization of the 25 ETEC strains (7 ETEC *tia*^+^/*leo*A^+^ strains, 14 ETEC *tib*A^+^ strains and one ETEC *tia*^+^/*tib*A^+^ strain) revealed significant genotypic diversity. Furthermore, the group of ETEC strains that possess both *tia* and l*eo*A genes shows a distinct distribution of toxicity genes: 57.1% carry *lt*, and 42.85% carry *st*. The predominant group, *tia*-negative but *tib*A-positive and *leo*A-negative, showed a mixed virulence profile, with 46.66% of strains carrying both toxins. In comparison, 33.33% have *st* and 20% have *lt* independently. For instance, *tia*-negative ETEC strains require a broader array of toxins (both LTs and STs) to ensure that their relatively short duration in the intestine is sufficient to cause disease. In addition, *tia*-positive strains can produce only one toxin, as their presence in intestinal tissue is more stable and persistent. These bacteria, which lack invasion factors such as Tia, increase their chemical arsenal when both LTs and STs are used to induce significant fluid secretion. From a functional perspective, another author reported that LeoA acts as a GTPase, facilitating the secretion of LTs through outer membrane vesicles (Brown and Hardwidge, 2007). A systematic review by Isidean et al. (2011) indicated that strains producing both toxins (LTs/STs) are often linked to more severe diarrhea episodes and greater dehydration, especially in children.

Phenotypic heterogeneity in biofilm formation among *tibA*-positive ETEC strains demonstrates multifactorial complexity. Notably, 66.67% of these strains exhibited low biofilm production, indicating that the presence of the tibA gene alone does not ensure robust biofilm formation. The efficiency of this system depends on posttranslational glycosylation by TibC (Sherlock et al., 2005), synergistic interactions among multiple fimbriae (Espert et al., 2011), and the coexpression of CFAs (Cruz-Córdova et al., 2014). Although *in vitro* assays reveal variability, the intestinal environment may facilitate increased biofilm formation (Sauvaitre et al., 2022), underscoring the clinical relevance of these interactions *in vivo*.

Future studies are needed to elucidate the roles of the Tia, TibA, and LeoA afimbrial adhesins in adherence, invasion, and biofilm formation. The construction of isogenic mutants is essential for determining their specific contribution to these phenotypes and for identifying the receptors involved in recognition of these adhesins.

## 5 Conclusions

ETEC strains harboring the noncanonical virulence genes *tia, tib*A, and *leo*A exhibited MDR profiles and the ability to adhere to and invade the HT-29 and HuTu80 cell lines, as well as to form biofilms. Although this study assessed only the presence of these genes, the observed phenotypic characteristics of ETEC strains indicate their potential for adaptation to different environments and highlight their genotypic background as a possible public health concern.

## Data Availability

The raw data supporting the conclusions of this article will be made available by the authors, without undue reservation.
