# Supplementary material for: Prevalence of noncanonical virulence factors in the adherence and invasion by enterotoxigenic Escherichia coli in the small and large intestines
Source: Front Cell Infect Microbiol. 2026 Aug 24;16:1892042. doi: 10.3389/fcimb.2026.1892042 (PMC13547076; doi:10.3389/fcimb.2026.1892042)
Supplement: Supplementary file 1 [file Table2.docx]

**Supplementary Table 2.** Endpoint PCR amplification conditions for identifying noncanonical genes in ETEC strains.

| **Genes** |  | **Amplification (25 cycles)** | | | |  |
| --- | --- | --- | --- | --- | --- | --- |
|  | **Initial Denaturing**  **(1 cycle)** | **Denaturation** | **Annealing (Tm)** | **Extension** | | **Final extension**  **(1 cycle)** |
| ***tia*** | 95°C, 1 min | 94°C, 30 s | 54°C, 30 s | | 72°C, 1 min | 72°C, 5 min |
| ***tiba*** | 95°C, 1 min | 94°C, 30 s | 54°C, 30 s | | 72°C, 1 min | 72°C, 5 min |
| ***leoA*** | 95°C, 1 min | 94°C, 30 s | 54°C, 30 s | | 72°C, 1 min | 72°C, 5 min |
| ***eatA*** | 95°C, 1 min | 94°C, 30 s | 49.5°C, 1 min | | 72°C, 2 min | 72°C, 5 min |
| ***etpA*** | 95°C, 1 min | 94°C, 30 s | 54°C, 1 min | | 72°C, 2 min | 72°C, 5 min |
| ***etpB*** | 95°C, 1 min | 94°C, 30 s | 53°C, 1 min | | 72°C,1 min | 72°C, 5 min |

s = seconds and min = minutes
